# Fungal Communities in Rhizosphere Soil under Conservation Tillage Shift in Response to Plant Growth

**DOI:** 10.3389/fmicb.2017.01301

**Published:** 2017-07-11

**Authors:** Ziting Wang, Tong Li, Xiaoxia Wen, Yang Liu, Juan Han, Yuncheng Liao, Jennifer M. DeBruyn

**Affiliations:** ^1^College of Agronomy, Northwest A&F University Yangling, China; ^2^Department of Biosystems Engineering and Soil Science, The University of Tennessee Knoxville, TN, United States

**Keywords:** conservation tillage, rhizosphere soil, fungal community, plant growth, structural equation model

## Abstract

Conservation tillage is an extensively used agricultural practice in northern China that alters soil texture and nutrient conditions, causing changes in the soil microbial community. However, how conservation tillage affects rhizosphere and bulk soil fungal communities during plant growth remains unclear. The present study investigated the effect of long-term (6 years) conservation (chisel plow, zero) and conventional (plow) tillage during wheat growth on the rhizosphere fungal community, using high-throughput sequencing of the internal transcribed spacer (ITS) gene and quantitative PCR. During tillering, fungal alpha diversity in both rhizosphere and bulk soil were significantly higher under zero tillage compared to other methods. Although tillage had no significant effect during the flowering stage, fungal alpha diversity at this stage was significantly different between rhizosphere and bulk soils, with bulk soil presenting the highest diversity. This was also reflected in the phylogenetic structure of the communities, as rhizosphere soil communities underwent a greater shift from tillering to flowering compared to bulk soil communities. In general, less variation in community structure was observed under zero tillage compared to plow and chisel plow treatments. Changes in the relative abundance of the fungal orders Capnodiales, Pleosporales, and Xylariales contributed the highest to the dissimilarities observed. Structural equation models revealed that the soil fungal communities under the three tillage regimes were likely influenced by the changes in soil properties associated with plant growth. This study suggested that: (1) differences in nutrient resources between rhizosphere and bulk soils can select for different types of fungi thereby increasing community variation during plant growth; (2) tillage can alter fungal communities' variability, with zero tillage promoting more stable communities. This work suggests that long-term changes in tillage regimes may result in unique soil fungal ecology, which might influence other aspects of soil functioning (e.g., decomposition).

## Introduction

The rhizosphere, which is the volume of soil surrounding the plant root, is influenced by root activities such as exudation of reactive carbon compounds and uptake of mobile nutrients and water (George et al., 2006; Hartmann et al., 2008). Roots have evolved to adapt to their surrounding environment by optimizing their functional architecture to use resources in heterogeneous soils (Hinsinger et al., 2005; Pierret et al., 2007). Thus, the co-evolution of rhizosphere and plant roots play a major role in soil physical, chemical, and biological processes that sustain biodiversity, provide soil carbon sequestration, and cycle nutrients in natural and agricultural systems (Hinsinger et al., 2009; Lambers et al., 2009). The plant-rhizosphere system also affects the biomass and activity of soil microorganisms that is generally enhanced due to root exudates (Raaijmakers et al., 2009). Different soil types harbor particular indigenous microorganisms that control the influence of plant root activity on rhizosphere microbial communities (Singh et al., 2007; Berg and Smalla, 2009). Plant species releasing root exudates are thought to select for rhizosphere microbial populations that respond with chemotaxis and fast growth (Hartmann et al., 2009). Plant growth also affects the composition of the rhizosphere microbial community, as root exudates change during the plant's life cycle and seasonal environment responses (Baetz and Martinoia, 2014). However, most studies have focused on the rhizosphere bacterial communities, and only a few have focused on fungal communities in the plant rhizosphere.

Soil fungal communities play essential roles in biogeochemical cycles, organic matter decomposition, plant growth, and disease development and control (Raaijmakers et al., 2009). Rhizosphere fungi are closely linked to plant health and growth, owing to their roles in antagonizing pathogens, decomposing plant residues, and providing nutrients (Ehrmann and Ritz, 2014). Variation in the fungal community of the rhizosphere is suggested to be plant-dependent because roots release several organic compounds that contribute to a unique rhizosphere nutrient pool, which is accessible to soil microorganisms (Klaubauf et al., 2010; Jiang et al., 2012; Han et al., 2016). Soil physical and chemical properties are known to be significantly correlated with changes in the rhizosphere fungal community (Schappe et al., 2017). Soil texture highly affects the organic carbon content and consequently determines plant rhizosphere microbial communities (Singh et al., 2007; Wang et al., 2009). Soil enzyme activity, particularly the hydrolysis of immobilized complex biomolecules as a strategy for nutrient acquisition, is strongly associated with rhizosphere fungal communities (Welc et al., 2014). Thus, improving the knowledge on rhizosphere fungal communities is expected to result in a better understanding of their roles in soil ecosystems.

Conservation tillage is extensively used in the dryland regions of northern China, which account for ~56% of the nation's total land areas. In these regions, crop production is constrained by multiple factors, including adverse weather and topography, limited water resources, and exhaustion of available soil water and nutrients due to conventional agricultural practices (Wang et al., 2007). Conservation tillage can improve the soil structure, conserve soil water, increase soil nutrient levels, substantially increase the efficiency of water-use for crops, and increase crop yields (Zhang et al., 2012). Our previous study indicated that conservation tillage affects crop residue decomposition, thereby altering soil organic carbon (SOC) content and leading to changes in the distribution patterns of the soil fungal community (Wang et al., 2016a). Using indirect techniques, a few studies have shown that conservation tillage modifies the rhizosphere environment, changing substrate utilization and arbuscular mycorrhizal (AM) fungal communities (Lupwayi et al., 1998; Mirás-Avalos et al., 2011). However, these studies could not clearly and accurately delineate the differences between the rhizosphere fungal community of soils subject to conservation and conventional tillage. In particular, information on how conservation tillage influences the rhizosphere fungal community responses to winter wheat growth is quite limited.

The present study used quantitative PCR (qPCR) and Illumina MiSeq (Illumina, San Diego, CA, USA) deep sequencing to characterize fungal communities within the rhizosphere and bulk soil of a cropland with a wheat–maize rotation. To further understand the dynamics of fungal communities during plant growth, winter wheat root systems were sampled at two growth stages. We assumed that tillage influenced rhizosphere ecology, altering the rhizosphere fungal community as a consequence of changes in the surrounding soil environment in response to plant growth. The present investigation aimed to determine: (1) how rhizosphere and bulk soil fungal communities are affected by the growing roots of winter wheat; (2) if long-term tillage influences the patterns of rhizosphere and bulk soil fungal communities' response to plant growth.

## Materials and methods

### Study site

This study was performed at Northwest A&F University, Yanglin, Shaanxi, China (34°17′N, 108°04′E), which is 521 m above sea level. The mean annual precipitation in this region is 633 mm, and the average annual temperature is 13.2°C. The experimental area was located in the Guanzhong Plain, which belongs to the drylands of northern China (Wang et al., 2016b), where a long-term trial began in 2009. Before this year, the experimental area was managed using rotary cultivation. Winter wheat (cv. Shaanmai 139) was sown over the residues of maize (cv. Shaandan 609) on October 18, 2014 using wheat drills. Urea fertilizer, with nitrogen (N) content >46%, and calcium phosphate fertilizer, with 16% phosphorous (P), were applied to all treatments (750 kg·ha^−1^) at the time of soil preparation.

### Tillage treatment and soil sampling

Experimental treatments combined three tillage methods and residue retention in wheat–maize rotation croplands. The main characteristics of conservation (chisel plow and zero) and conventional (plow) tillage treatments are described in Wang et al. (2016a).

Sampling was conducted at the tillering (vegetative) stage (November 21, 2014, after 34 days of snow) and at the flowering (reproductive) stage (May 2, 2015, after 196 days of snow). Rhizosphere soil was sampled from randomly selected wheat plants showing similar characteristics; their roots were then vigorously shaken to remove the soil not tightly adhering to them (Smalla et al., 2001). Bulk soil samples were collected away from plant roots, at 0–20 cm depth, using a standard soil corer. All samples were sieved through a 2-mm mesh to eliminate large rocks and roots. Each composite soil sample (rhizosphere or bulk soil) was homogenized and stored at 4°C for less than 24 h until DNA extraction.

### Soil physicochemical analysis

The physical and chemical properties of the soil were analyzed in the laboratory. SOC, total N, and texture were measured as previously described (Zhao et al., 2014). Soil moisture was measured gravimetrically. Urease and invertase activities were assayed in soil samples (5 g), after adding an appropriate substrate and incubating the soil and substrate mixture for 24 h at 37°C and at the optimal pH for each enzyme, as described by Gu et al. (2009).

### DNA extraction, PCR amplification, and illumina sequencing

Microbial DNA was extracted from fresh soil (3 replicates × 1 g) using the E.Z.N.A. Soil DNA Kit (Omega Bio-tek, Inc., Norcross, GA, USA), according to the manufacturer's instructions. The concentration and quality of the extracted DNA were determined using a NanoDrop 2000 spectrophotometer (Thermo Scientific, Wilmington, DE, USA). The fungal internal transcribed spacer-1 (ITS-1) region was amplified from each sample using primers ITS1F (5′-ACTTGGTCATTTAGAGGAAGTAA-3′) and ITS2 (5′-BGCTGCGTTCTTCATCGATGC-3′) (Mukherjee et al., 2014), which provide a comprehensive coverage with the highest taxonomical accuracy for fungal sequences (Mello et al., 2011). The reverse primer contained a 6-bp error-correcting barcode unique to each sample. The PCR protocol used to amplify ITS-1 has been described previously (Mukherjee et al., 2014), and sequencing was performed on an Illumina MiSeq PE300 instrument obtained from Majorbio BioPharm Technology Co., Ltd. (Shanghai, China).

The software FLASH was used to merge paired sequence reads generated from the original DNA fragments (Caporaso et al., 2010). The sequences were further analyzed using USEARCH v 5.2.32 to filter and denoise the data by clustering sequences with <3% dissimilarity. The QIIME pipeline was used to select ITS-1 operational taxonomic units (OTUs) by combining reads of clustered OTUs with 97% similarity (Edgar, 2010). The ITS-1 sequences obtained in this study were deposited in the National Center for Biotechnology Information (NCBI) Sequence Read Archive (SRA) database under accession SRP080901.

### Quantitative PCR

The relative abundances of the fungal 18S rDNA gene copies were measured by qPCR using the fungi-specific primer pair FR1 (5′-ANCCATTCAATCGGTANT-3′) and FF390 (5′-CGATAACGAACGAGACCT-3′) (Chemidlin Prevost-Boure et al., 2011). The 20-μL qPCR mixture contained 10 μL EvaGreen 2 × qPCR MasterMix (Applied Biological Materials Inc., Richmond, BC, Canada), 0.3 μM each primer (final concentration), and fungal, environmental, or standard DNA templates (10–20 ng μL^−1^ per reaction). The qPCR experiments were performed in a Bio-Rad C1000/CFX96 Thermocycler (Bio-Rad, Hercules, CA, USA) using an initial denaturation at 95°C for 10 min followed by 29 cycles at 95°C for 15 s, 50°C for 30 s, and 70°C for 60 s. The temperature was then increased from 65°C to 95°C at 0.5°C s^−1^ to perform a melting curve analysis of the PCR products. All qPCRs were run in triplicate for each DNA template. The abundances of fungal 18S rDNA genes were quantified using standard curves generated from 10-fold serial dilutions of cloned full-length copies of the 18S rDNA gene. Amplification efficiency ranged from 91 to 97%, and the R^2^ values of the standard curves ranged from 0.996 to 0.999.

### Statistical and bioinformatics analyses

Alpha diversity was estimated using the Shannon and the Simpson diversity indices. Estimation of the beta diversity and phylogenetic community comparisons were performed using weighted and unweighted UniFrac distance matrices. Taxonomic compositions were determined based on the relative abundances of dominant orders within Ascomycota, Basidiomycota, and Zygomycota. Correlations between the soil bacterial community structure and soil characteristics were determined using Mantel tests with 999 permutations.

Both the ANOVA and the Spearman's rank correlations between abundant phyla and soil properties were performed in SPSS 22.0 (SPSS Inc., Chicago, IL, USA). The principal coordinate analysis (PCoA) based on the Bray distance, the non-metric multidimensional scaling (NMDS) based on weighted and unweighted UniFrac distances, PERMANOVA, SIMPER, and Mantel tests were performed using the “vegan” package in the R v 3.20 statistical environment (Oksanen et al., 2013). All tests considered *P* < 0.05 as the significance threshold.

### Structural equation model

Two path analyses were conducted to measure the direct and indirect effects of tillage, plant growth, and rhizosphere on soil fungal communities. In path analysis, a structural equation model (SEM) is designed to feature variables and to hypothesize causal relationships among these variables in a path diagram (Fanin and Bertrand, 2016). Here, we considered plant growth as the result of dry matter accumulation associated with yield–trait relationships of wheat in China (Meng et al., 2013). Considering that plant roots release a great variety of compounds that distinguish the soil surrounding the roots from bulk soils, we used the carbon (C) partitioned below the ground to represent the rhizosphere effects in the SEM (Jones et al., 2009). For tillage treatments, the fractal characterization of soil aggregation and fragmentation were thought to quantify the soil disturbance caused by tillage (Perfect and Blevins, 1997). The degree of soil disturbance distinguished the three tillage treatments (plow tillage > chisel plow tillage > zero tillage); differences in net fixed carbon distinguished rhizosphere from bulk soil (rhizosphere > bulk); dry matter accumulation weight was considered the winter wheat growth from the tillering to the flowering stage (flowering > tillering).

The adequacy of the model was assessed by χ^2^ tests (*P* > 0.05) and by the calculation of the root mean square error of approximation (RMSEA) (values <0.05). These statistical tests were performed in R using the “lavaan” package.

## Results

### Plant growth shaped fungal alpha diversity and abundance in rhizosphere soil

Shannon and Simpson indices were used as estimates of fungal alpha diversity. The ANOVA results showed that tillage treatments (For Shannon/Simpson indices: *F* = 8.476/4.820, *P* = 0.005/0.029) had a stronger effect on the bulk soil fungal alpha diversity than plant growth (*F* = 1.070/0.802, *P* = 0.321/0.388). In contrast, fungal alpha diversity in the rhizosphere soil was significantly influenced by both plant growth (*F* = 7.395/8.707, *P* = 0.019/0.012) and tillage (*F* = 7.131/6.864, *P* = 0.009/0.010). In addition, soil fungal alpha diversity was significantly influenced by tillage in the tillering stage and by rhizosphere or bulk soil in the flowering stage (Table S1). Different variation patterns were found among the three tillage treatments. Plow tillage showed a larger difference in fungal alpha diversity between rhizosphere and bulk soils than conservation (chisel plow and zero) tillage (Table 1). Fungal alpha diversity was significantly correlated with SOC, invertase activity, soil texture, and moisture in zero tillage, whereas in plow tillage changes in fungal alpha diversity were significantly correlated to soil invertase, urease, and soil texture (Table S1).

**Table 1:** Soil physicochemical properties, qPCR, and alpha-diversity ranks according to tillage and temporal-spatial treatments.

**Tillage**	**Treatment**	**Soil texture (%)**	**Soil moisture (%)**	**TN (g/kg)**	**SOC (g/kg)**	**Urease (mg/g)**	**Invertase (mg/g)**	**qPCR (log_10_)**	**Alpha-diversity**	
									**Shannon**	**Simpson**
PT	TB	0.043 NS b	15.8 B a	0.760 NS ns	7.164 B ab	7.802 NS b	2.880 B d	6.708 A ns	4.017 B ab	0.049 A bc
	FB	0.013 B c	14.2 NS ab	0.785 NS ns	7.500 NS a	8.116 B b	5.307 C c	6.513 NS ns	4.260 NS a	0.037 NS c
	TR	0.087 NS a	15.6 B a	0.797 B ns	6.564 B ab	16.908 NS a	13.295 NS a	6.757 NS ns	3.677 B bc	0.068 A ab
	FR	0.095 B a	11.8 NS b	0.795 C ns	6.219 C b	11.633 AB b	11.069 B b	6.800 NS ns	3.483 NS b	0.083 NS a
CPT	TB	0.043 NS b	17.0 A a	0.809 NS c	9.293 A ab	8.225 NS b	3.743 A d	6.445 B c	3.903 B ns	0.058 A ab
	FB	0.037 A b	14.9 NS b	1.059 NS bc	8.345 NS b	9.420 AB b	8.796 A c	6.549 NS bc	4.153 NS ns	0.046 NS b
	TR	0.098 NS a	17.3 A a	1.345 A ab	8.327 A b	12.986 NS a	13.924 NS b	6.932 NS a	4.070 AB ns	0.054 AB ab
	FR	0.117 A a	11.4 NS c	1.483 A a	10.401 A a	9.333 B b	15.338 A a	6.702 NS ab	3.767 NS ns	0.068 NS a
ZT	TB	0.023 NS c	16.4 AB ab	1.037 NS bc	9.159 A ns	8.744 NS bc	3.456 A c	6.485 AB ns	4.550 A a	0.027 B b
	FB	0.020 B c	14.6 NS b	0.987 NS c	8.432 NS ns	11.522 A c	7.523 B b	6.517 NS ns	4.313 NS a	0.038 NS b
	TR	0.070 NS a	17.8 A a	1.256 A a	8.749 A ns	15.150 NS a	13.458 NS a	6.627 NS ns	4.247 A a	0.038 B b
	FR	0.054 C b	11.8 NS c	1.197 B ab	8.364 B ns	14.744 A ab	12.560 B a	6.613 NS ns	3.873 NS b	0.060 NS a

a *Values are mean of three soil samples. Soil texture, Sand/(Clay+Silt); SOC, soil organic carbon; TN, total nitrogen*.

b *PT, Plow tillage; ZT, Zero tillage; CPT, Chisel plough tillage; TB, tillering bulk soil; FB, flowering bulk soil; TR, tillering rhizosphere; FR, flowering rhizosphere*.

c *Different letters indicate significant differences (ANOVA, P < 0.05, Tukey's HSD post-hoc analysis) among tillage (capital letter) and temporal-spatial treatments (small letter)*.

Soil fungal abundance was significantly different between rhizosphere and bulk soils in the tillering (*F* = 9.246, *P* = 0.010) and flowering (*F* = 8.745, *P* = 0.012) stages. In the rhizosphere soil, fungal abundance significantly increased in chisel plow tillage, as opposed to that registered in plow and zero tillage (Table 1). Spearman correlation showed that soil invertase (*R* = 0.601, *P* = 0.001) and urease (*R* = 0.754, *P* = 0.009) activities and total nitrogen (TN; *R* = 0.636, *P* = 0.028) were significantly related to fungal abundance in chisel plow tillage, but only soil invertase correlated with fungal abundance in zero tillage (*R* = 0.648, *P* = 0.017) and plow tillage (*R* = 0.580, *P* = 0.048).

### Effect of plant growth on rhizosphere soil fungal beta diversity

**Figure 1:**
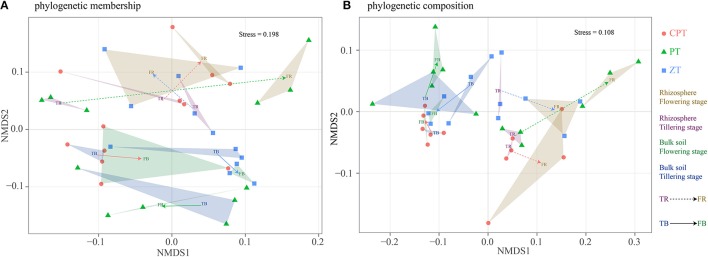
Fungal community structure indicated by non-metric multi-dimensional scaling (NMDS) plots of weighted **(A)** and unweighted **(B)** pairwise UniFrac distances. PT = Plow tillage (*circle*); ZT = Zero tillage (*square*); CPT = Chisel plow tillage (*triangle*).

Overall, 1,564,488 quality sequences and a mean of 16,826 sequences per sample were obtained across all soil samples. The phylogenetic analysis of community membership and composition was performed using unweighted and weighted UniFrac distances, respectively, and the changes in the fungal phylogenetic structure according to the plant growth stage were evaluated by NMDS analyses (Figures 1A,B). Changes in the phylogenetic structure (membership and composition) of fungal communities in the tillering and flowering stages were generally much stronger in the rhizosphere (PERMANOVA: R^2^ = 0.147/0.304, *P* = 0.001/0.001) than in bulk soil (PERMANOVA: R^2^ = 0.082/0.081, *P* = 0.049/0.038). However, bulk soil fungal phylogenetic structure was significantly influenced by tillage (PERMANOVA: R^2^ = 0.238/0.328, *P* = 0.001/0.001). Phylogenetic composition (PERMANOVA: R^2^ = 0.549, *P* = 0.001; Figure 1B) had more clear clusters than phylogenetic membership (PERMANOVA: R^2^ = 0.309, *P* = 0.001; Figure 1A). Rhizosphere and bulk soil fungal phylogenetic structure also varied according to plant growth among the three tillage treatments (Figure 1). Significantly lower variations between rhizosphere and bulk soil fungal phylogenetic membership and plant growth were found in zero tillage compared to plow and chisel plow tillage (Figure 1A). In addition, zero tillage led to lower variation in the fungal phylogenetic composition between tillering and flowering stages compared to the other tillage treatments (Figure 1B). Soil properties had significantly different relationships with fungal phylogenetic membership and composition among the three tillage treatments (Table 2). Soil physical structure (texture and moisture; Mantel test: *P* < 0.05) and invertase activity (Mantel test: *P* < 0.05) were the main factors determining the variation of fungal phylogenetic structure (membership and composition; Table 2). Additionally, SOC (Mantel test: *P* < 0.05) and total nitrogen (Mantel test: *P* < 0.05) influenced fungal phylogenetic structure in chisel plow and plow tillage, respectively (Table 2).

**Table 2:** Mantel test results showing the significant correlation between soil properties and fungal beta diversity.

**Beta diversity**	**Tillage^b^**	**Soil texture^a^**	**Soil moisture**	**TN**	**SOC**	**Urease**	**Invertase**
Phylogenetic membership	PT	0.370^*^	0.503^**^	−0.069^ns^	0.287^*^	0.136^ns^	0.349^*^
	CPT	0.532^**^	0.365^**^	0.252^*^	0.107^ns^	−0.039^ns^	0.466^**^
	ZT	0.167^ns^	0.444^**^	0.137^ns^	0.023^ns^	0.137^ns^	0.226^*^
Phylogenetic composition	PT	0.572^**^	0.525^**^	−0.022^ns^	0.356^*^	0.020^ns^	0.455^**^
	CPT	0.769^**^	0.458^**^	0.400^*^	0.183^ns^	0.131^ns^	0.547^**^
	ZT	0.307^*^	0.228^ns^	0.205^ns^	−0.069^ns^	−0.036^ns^	0.294^*^
Taxonomic composition	PT	0.297^*^	0.696^**^	0.030^ns^	0.299^*^	−0.118^ns^	0.153^ns^
	CPT	0.613^**^	0.673^*^	0.322^*^	0.359^*^	0.037^ns^	0.420^*^
	ZT	0.052^ns^	0.211^ns^	0.005^ns^	−0.050^ns^	−0.132^ns^	0.126^ns^

a *Values are mean of three soil samples. Soil texture, Sand/(Clay+Silt); SOC, soil organic carbon; TN, total nitrogen*.

b *PT, Plow tillage; ZT, Zero tillage*.

* *0.01 < P < 0.05*;

** *P < 0.01; ns, no significant P >0.05*.

**Figure 2:**
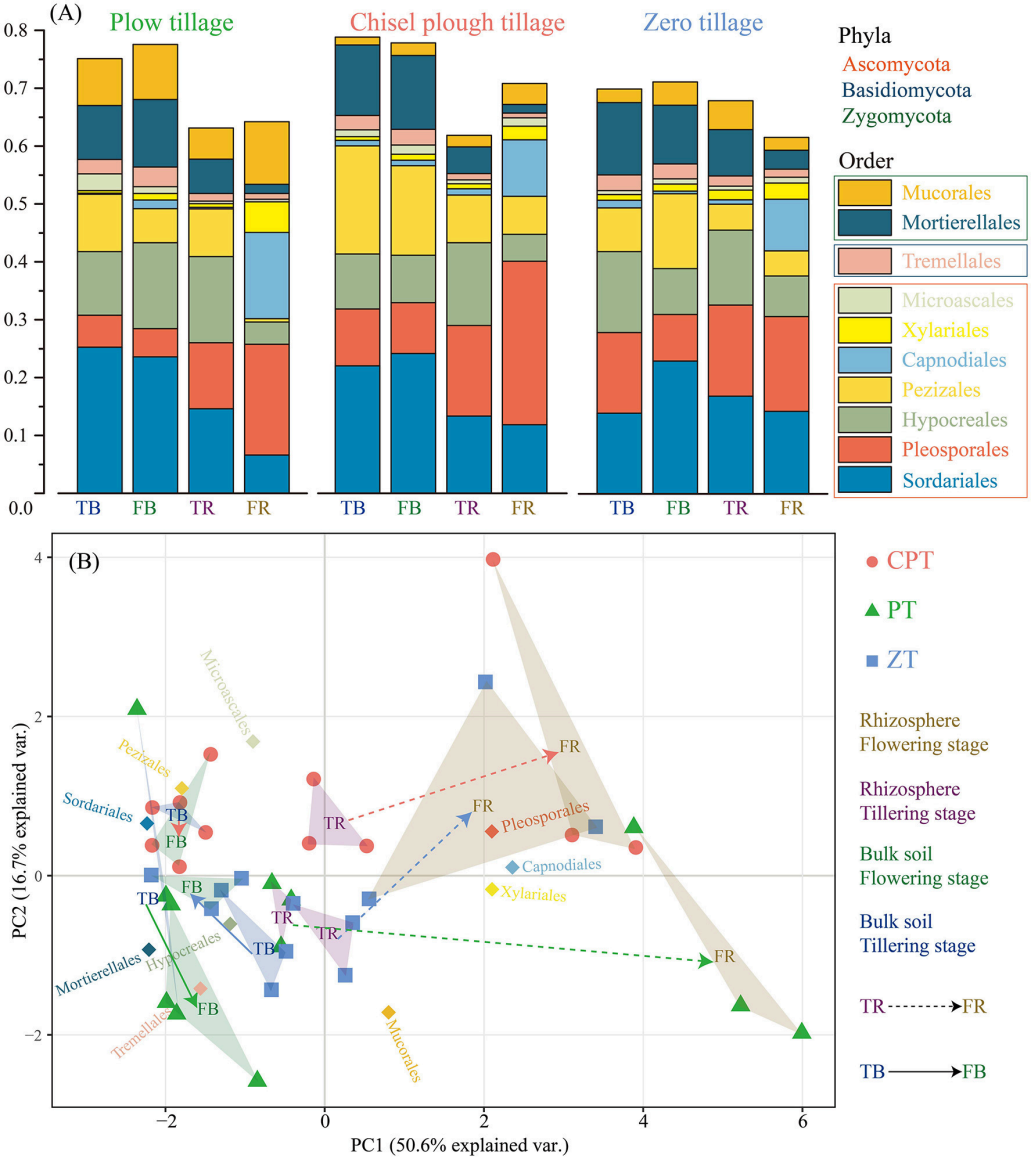
**(A)** Relative abundance of the dominant fungal orders in all soil samples combined and in each tillage treatment. **(B)** Principal Coordinate Analysis (PCoA) of abundant fungal orders.

Among all sequences, 82.2% were classified in bulk soil and 69.8% in rhizosphere soil. The dominant fungal phyla across all soil samples were Ascomycota (average 68.7%), Zygomycota (average 13.3%), and Basidiomycota (average 4.1%); at the order level, the fungal communities of all soil samples were dominated by Sordariales, Pleosporales, Hypocreales, Pezizales, Capnodiales, Xylariales, Microascales, Mortierellales, Mucorales, and Tremellales (Figure 2A). The PCoA based on the Bray distance conducted to assess the dynamics of rhizosphere and bulk soil fungal taxonomic composition over plant growth stages revealed that the composition of rhizosphere fungal communities differed significantly from bulk soil communities (PERMANOVA: R^2^ = 0.325, *P* = 0.001) in both tillering and flowering stages (Figure 2B). Bulk soil samples were clearly clustered and significantly separated according to tillage treatments (PERMANOVA: R^2^ = 0.308, *P* = 0.001), and plant growth stages rather than tillage treatment significantly influenced (PERMANOVA: R^2^ = 0.502, *P* = 0.001 vs. R^2^ = 0.122, *P* = 0.041; Figure 2B) fungal taxonomic composition in the rhizosphere soil. In addition, zero tillage samples were considerably closer to each other than chisel plow and plow tillage samples (Figure 2B). The relative abundance of fungal orders varied in the rhizome and bulk soil samples along plant growth, but showed different patterns among the three tillage treatments (Figure 2A, Table S2). Soil physical properties (texture/moisture) and invertase activity was significantly related to fungal taxonomic composition under plow tillage (Table 2); SOC and soil texture were the main factors distinguishing fungal communities under chisel plow and zero tillage (Table 2). Changes in the relative abundance of fungal orders (Capnodiales, Pleosporales, and Xylariales) contributed to the dissimilarities observed in the patterns of rhizosphere and bulk soil fungal taxonomic composition along plant growth (Table 3, Table S2).

**Table 3:** SIMPER analysis results showing the dominant fungal orders that contributed to the dissimilarity between rhizosphere and bulk soil and growth stages among tillage treatments.

**Stages compared**	**PT^a^**		**CPT**		**ZT**	
	**Order**	**%**	**Order**	**%**	**Order**	**%**
BT-BF^b^	**All**	**11.98**	**All**	**6.78**	**All**	**13.09**
	*Mucorales*	14.61	*Pezizales*	18.40	*Pezizales*	20.54
	*Pezizales*	14.33	*Mucorales*	15.04	*Sordariales*	17.38
	*Capnodiales*	13.45	*Mortierellales*	13.71	*Pleosporales*	14.91
RT-RF	**All**	**32.28**	**All**	**20.84**	**All**	**16.59**
	*Capnodiales*	23.53	*Capnodiales*	22.40	*Capnodiales*	28.17
	*Pezizales*	15.30	*Hypocreales*	17.54	*Mortierellales*	15.34
	*Hypocreales*	13.88	*Pleosporales*	14.49	*Hypocreales*	12.84
TB-TR	**All**	**13.51**	**All**	**15.8**	**All**	**9.88**
	*Sordariales*	18.78	*Pezizales*	20.53	*Pezizales*	21.75
	*Pleosporales*	17.19	*Mortierellales*	18.47	*Mucorales*	16.35
	*Microascales*	13.42	*Sordariales*	14.49	*Mortierellales*	15.06
FB-FR	**All**	**35.57**	**All**	**27.75**	**All**	**21.63**
	*Capnodiales*	15.83	*Pleosporales*	17.91	*Capnodiales*	23.60
	*Sordariales*	14.33	*Mortierellales*	17.91	*Pezizales*	15.26
	*Pleosporales*	13.19	*Capnodiales*	16.38	*Mortierellales*	15.09

a *PT, plow tillage; CPT, chisel plough tillage; ZT, zero tillage*.

b *BT, bulk-tillering stage; RT, rhizosphere-tillering stage; BF, bulk-flowering stage; RF, rhizosphere-flowering stage*.

### Structural equation models

**Figure 3:**
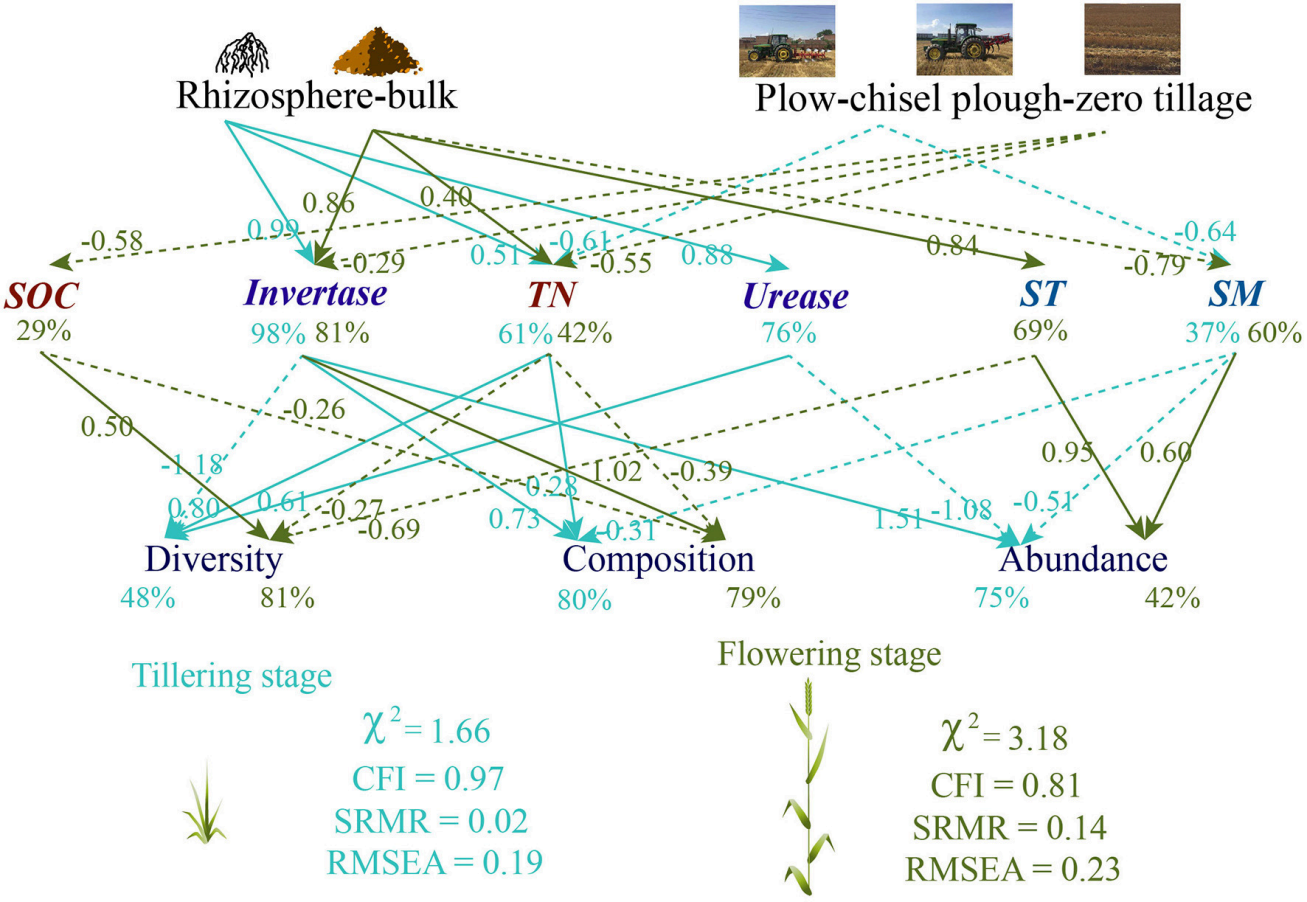
Path diagrams of the structural equation models for the relationship that tillage and plant growthon soil fungal diversity, abundance, and composition.

We hypothesized that changes in soil properties caused by tillage and rhizosphere would have a different effect on soil fungal diversity, abundance, and composition between plant growth stages. The SEM analyses were performed separately for plant growth stages and indicated that rhizosphere and tillage directly induced more changes in soil properties in the flowering stage (SOC: 29.2%, invertase: 80.5%, soil texture: 69.0%, TN: 42.4%, soil moisture: 60.1%) than in the tillering stage (urease: 76.3%, invertase: 98.4%, TN: 61.3%, soil moisture: 36.9%; Figure 3). Soil invertase activity was the main determinant of the soil fungal community in the tillering stage, whereas soil texture and invertase significantly influenced fungal diversity, composition, and abundance in the flowering stage (Figure 3).

**Figure 4:**
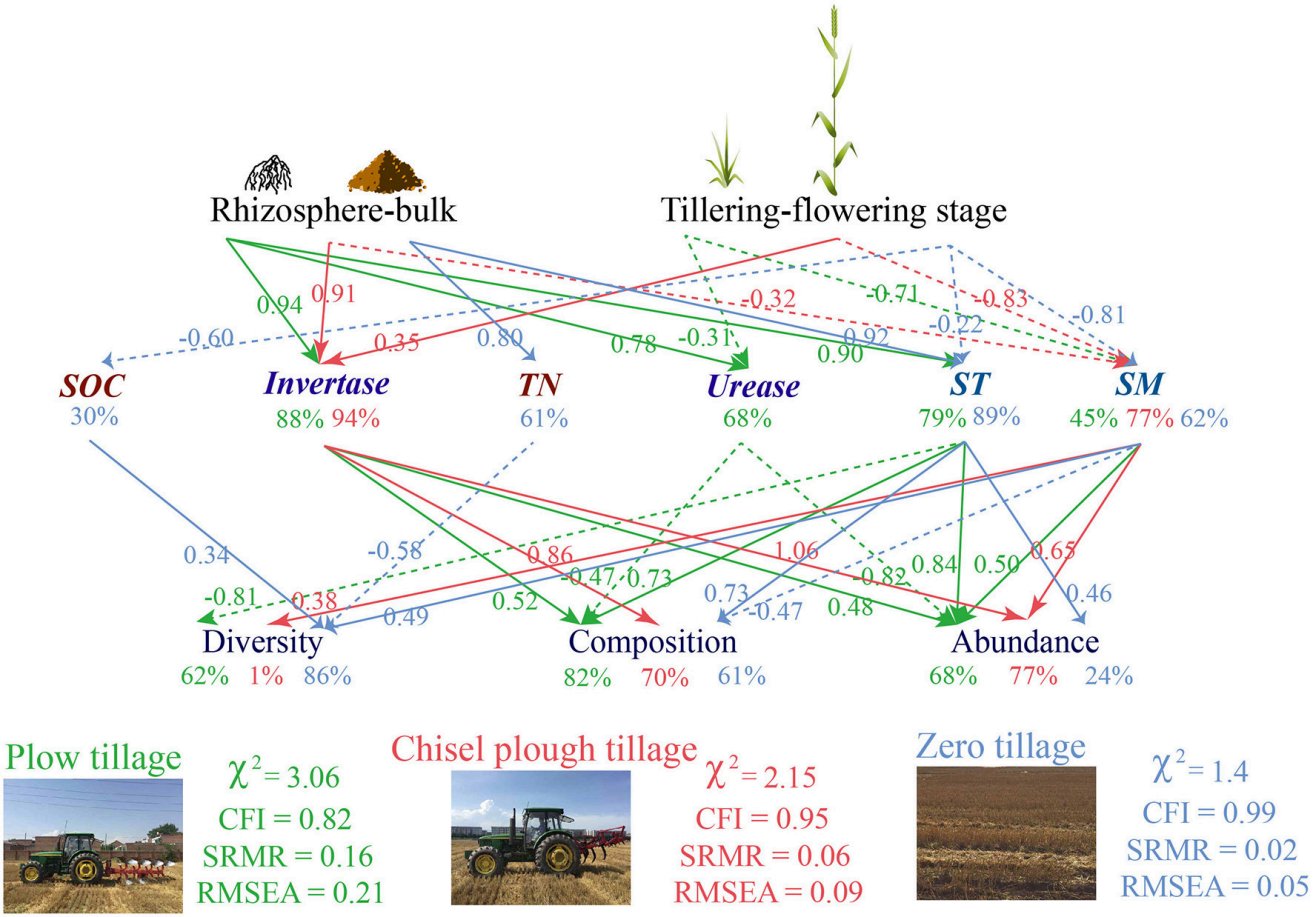
Path diagrams of the structural equation models for soil fungal community influenced by soil properties under the three tillage treatments in response to rhizosphere and plant growth.

A comparison of the three tillage treatments showed that the soil fungal community was differently influenced by changes in soil properties associated with plant growth (Figure 4). Soil enzyme activities and physical properties lead to changes in fungal communities in chisel plow (invertase: 94.2%, soil moisture: 77.2%) and plow tillage (urease: 68.0%, invertase: 88.2%, soil texture: 79.1%, soil moisture: 45.4%), whereas soil total nutrients (SOC: 29.8%, TN: 61.1%), and physical properties (soil texture: 89.1%, soil moisture: 62.3%) influenced fungal communities in zero tillage soils (Figure 4). Compared to plow tillage, fungal diversity (1.2%) and abundance (23.9%) had a lower and non-significant influence on the variation noted in chisel plow and zero tillage, respectively (Figure 4).

## Discussion

### Influence of plant growth on rhizosphere fungal community

The present study investigated the influence of plant growth on rhizosphere and bulk fungal communities under three tillage treatments. Larger variations in fungal alpha diversity between rhizosphere and bulk soils were identified at the flowering stage than at the tillering stage. This was due to the opposite changes in soil fungal alpha diversity in rhizosphere and bulk soils along plant growth (Table 1); notably, fungal alpha diversity was more strongly influenced by plant growth in the rhizosphere than in bulk soils. Soil enzyme activities and physical structure, as major factors affecting microbial communities, were highly correlated with both Shannon and Simpson indices, which can be explained by rhizosphere fungal alpha diversity being mostly shaped by plant growth stages (Nannipieri et al., 2003; Bell et al., 2009).

Trends in the changes of fungal phylogenetic structure (membership and composition) were consistent with variations in the fungal alpha diversity under the three tillage treatments. Fungal phylogenetic structure is more sensitive to plant growth in the rhizosphere than in bulk soils because the latter is highly stable (Houlden et al., 2008). In addition, plant growth has a greater impact on fungal phylogenetic composition than on phylogenetic membership because similar plant species select similar plant-driven microbial species by influencing the available nutrients in the surrounding soil (Hartmann et al., 2009). Changes in fungal phylogenetic membership and composition showed a significant relationship with soil invertase activities (Table 2). Higher soil invertase activity along roots depends on the root system, implying that the rhizosphere soil environment was strongly affected by plant root activities, thus causing larger variations in the fungal phylogenetic structure between tillering and flowering stages in rhizosphere soils (Welc et al., 2014; Razavi et al., 2016). In contrast, lower soil invertase activity in bulk soils reflects the weak influence of root activity on bulk soil fungal microbial community, and therefore management (i.e., tillage) is the main factor determining bulk soil environment during plant growth (Niu et al., 2015; Banerjee et al., 2016).

Compared to the tillering stage, fungal taxonomic composition significantly differed between rhizosphere and bulk soils at the flowering stage, under the three tillage treatments. Due to the significant increase in the relative abundance of Capnodiales, Pleosporales, and Xylariales, fungal taxonomic composition was more affected by plant growth stage in the rhizosphere than in bulk soil. Fungi within these orders (phylum Ascomycota) grow quickly and become dominant at the flowering stage because they are able to immediately use carbon resources released by roots (Hannula et al., 2012). Because the dominant Sordariales and Mortierellales are considered to be primary straw residue decomposers in arable soils, and the fungal taxonomic composition in bulk soils was likely driven by residue decomposition processes in crop residuals (Ma et al., 2013). The taxonomic composition distinguished tillering from flowering stages due to fungal proportion changes in the early stages of residue decomposition. Bastian et al. (2009) showed that the microbial succession on fresh organic residue incorporated in soil was dominated by copiotrophs and r-strategists in the early stages of decomposition.

According to the SEM, soil and rhizosphere fungal communities were more differentiated at the flowering than at the tillering stage due to the influence of soil properties. In particular, SEM revealed that increasing variations in the fungal community between rhizosphere and bulk soils during plant growth were caused by changes in soil nutrient status due to an increase in root exudates (Houlden et al., 2008). Consequently, different fungi thereby increasing the variations in fungal communities between rhizosphere and bulk soils during plant growth.

### Tillage treatments shaped rhizosphere fungal communities

We found higher variations in both Shannon and Simpson indices under conventional (plow) tillage than under conservation (chisel plow, zero) tillage. In addition, fungal alpha diversity was significantly and uniquely correlated to soil properties under each tillage treatment. Zero tillage had the highest Shannon diversity value associated with lower variations in SOC and soil invertase activity, likely due to the build-up of labile C pools under conservation tillage (Chen et al., 2009). Relatively stable SOC and invertase activity indicated that zero tillage was preserving soil nutrient status, promoting fungal diversity (Plaza et al., 2013).

Although plant growth increased the distinction of fungal phylogenetic structure between rhizosphere and bulk soils among the three tillage treatments, the least variability in phylogenetic structures were observed under zero tillage. These results can be explained by lower variations of soil physical structure, which is significantly correlated with changes in the fungal phylogenetic membership and composition under zero tillage. Soil physical structure is important in terms of moisture retention, organic C storage, and cation exchange; all of which have been demonstrated to shape microbial communities (Chodak et al., 2016). In contrast to zero tillage, plow tillage, which is a conventional management practice that disturbs soil density, results in relatively low nutrient levels (here we observed low SOC and invertase activity), leading to a fungal phylogenetic structure that is more sensitive to plant growth and root activity (Yin et al., 2010; Wang et al., 2016a).

Rhizosphere fungal taxonomic composition was most balanced under conservation tillage at the flowering stage because the relative abundance of the major fungal orders (Capnodiales and Pleosporales) increased in similar proportion to plant growth. Capnodiales and Pleosporales, which belong to the class Dothideomycetes, have different strategies for breaking down cellulose in root-surrounding soils (Ohm et al., 2012), suggesting that plow tillage may alter nutrient use between the tillering and flowering stages in the rhizosphere more so than conservation tillage. At the tillering stage, zero tillage resulted in the greatest similarities between rhizosphere and bulk soil had more similar proportions of with respect to dominant fungal orders, which is mostly due to Sordariales preferring fine soils and decomposing residuals in environments with diverse nutrients, such as those associated with decomposing plant residues (Klaubauf et al., 2010; Ma et al., 2013; Wang et al., 2016a).

The three different SEMs based on the three tillage treatments revealed that changes in soil properties may directly influenced soil fungal community due to plant growth and root activity. Root activity and plant growth caused lower variations in soil texture under zero tillage, explaining the more stable soil fungal community, while soil invertase activity, strongly correlated to root exudates, was the most important soil variable determining the fungal community under plow and chisel plow tillage. Different patterns of direct and indirect effects on soil fungal community can be considered in relation to the biogeochemical processes occurring under the three tillage treatments, implying that long-term tillage may lead to a unique soil fungal ecology in response to biotic and abiotic factors (Fierer et al., 2009; Eisenhauer et al., 2015). Consequently, zero tillage, which produces less disturbance, establishes nutrient-rich conditions that increase the stability of soil fungal community responses to root activity and straw decomposition.

## Conclusion

In this study, we explored the changes in rhizosphere and bulk soil fungal communities during plant growth under three tillage treatments. Tillage is considered an anthropic agriculture management practice that can alter soil biota causing direct and indirect effects on crop growth and soil nutrient transformation (Brussaard et al., 2007). The results of the present study revealed that plant growth increased the discrimination of fungal communities between bulk and rhizosphere soil, mostly due to increases in relative abundance of Sordariales and Mortierellales in bulk soil, as they decompose crop residues, and increases in Capnodiales and Pleosporales in rhizosphere soil, which can grow quickly by using carbon components from plant roots in rhizosphere soil. This study also revealed that the changes were not consistent between tillage, therefore, tillage may contribute to unique soil fungal ecology. In this study, tillage altered the variability in fungal communities, with zero tillage promoting more stable communities, likely because the practice preserves soil physical structure and nutrient status. Future research should focus on the differences in carbon resources between rhizosphere and bulk soils under the three tillage practices, quantifying the relationship between organic carbon source utilization and fungal communities.

## Author contributions

ZW contributed to design of the experiments, data analysis, and manuscript writing; TL contributed to experimentation; XW, YaL, JH, YuL, and JD contributed to data interpretation, and manuscript preparation.

### Conflict of interest statement

The authors declare that the research was conducted in the absence of any commercial or financial relationships that could be construed as a potential conflict of interest.
