# Supplementary material for: Fungal Communities in Rhizosphere Soil under Conservation Tillage Shift in Response to Plant Growth
Source: Front Microbiol. 2017 Jul 11;8:1301. doi: 10.3389/fmicb.2017.01301 (PMC5504275; doi:10.3389/fmicb.2017.01301)
Supplement: Supplementary file 1 [file Table1.docx]

Table S1 Shown are strongly Spearman’s rank correlation coefficients for the fungal alpha diversity and soil proprieties.

| Tillage | Soil variable content retiesraceration of our the Conflict of Interest Statement the end of our text. Thank you for your consideration of our | R | *P* value |
| --- | --- | --- | --- |
| PT (Shannon/Simpson) | Urease | -0.648/0.673 | 0.023/0.017 |
|  | Invertase | -0.615/0.664 | 0.033/0.018 |
|  | Soil texture | -0.839/0.902 | 0.001/<0.001 |
| ZT  (Shannon/Simpson) | SOC | 0.670/-0.626 | 0.012/0.022 |
|  | Invertase | -0.615/0.456 | 0.025/0.117 |
|  | Soil texture | -0.626/0.538 | 0.022/0.058 |
|  | Soil moisture | 0.621/-0.615 | 0.024/0.025 |

Non-significant in CPT.
