# Supplementary material for: Fungal Communities in Rhizosphere Soil under Conservation Tillage Shift in Response to Plant Growth
Source: Front Microbiol. 2017 Jul 11;8:1301. doi: 10.3389/fmicb.2017.01301 (PMC5504275; doi:10.3389/fmicb.2017.01301)
Supplement: Supplementary file 2 [file Table2.docx]

Table S2 Soil taxonomic composition according to tillage and temporal-spatial treatments

| phylum | Order | Plow tillage | | | | Chisel plough tillage | | | | Zero tillage | | | |
| --- | --- | --- | --- | --- | --- | --- | --- | --- | --- | --- | --- | --- | --- |
|  |  | TB | FB | TR | FR | TB | FB | TR | FR | TB | FB | TR | FR |
| Ascomycota | Sordariales | 25.2%  a A | 23.6%  ab | 14.6%  bc | 6.6%  c | 22.0%  a AB | 24.2%  a | 13.3%  b | 11.9%  b | 13.8%  b B | 22.9%  a | 16.8%  ab | 14.2%  b |
|  | Pleosporales | 5.6%  c AB | 4.9%  c | 11.4%  b B | 19.1%  a B | 9.8%  b B | 8.8%  b | 15.7%  b A | 28.2%  a A | 13.9%  a A | 8.0%  b | 15.7%  a A | 16.4%  a B |
|  | Hypocreales | 11.0%  a | 14.8%  a B | 14.9%  a | 3.8%  b B | 9.5%  b | 8.2%  b A | 14.3%  a | 4.7%  c AB | 14.0%  a | 7.9%  b B | 13.0%  a | 7.0%  b A |
|  | Pezizales | 9.9%  a B | 5.9%  a A | 8.2%  a | 0.6%  b B | 18.7%  a A | 15.5%  a B | 8.2%  b | 6.6%  b A | 7.6%  ab B | 12.9%  a AB | 4.4%  b | 4.3%  b AB |
|  | Capnodiales | 0.2%  b | 1.5%  b A | 0.3%  b B | 14.9%  a | 0.9%  b | 0.9%  b AB | 1.1%  b A | 9.8%  a | 1.3%  b | 0.4%  b C | 0.8%  b AB | 8.9%  a |
|  | Xylariales | 0.5%  b B | 1.1%  b | 0.6%  b B | 5.3%  a | 0.6%  B | 1.1% | 0.9%  B | 2.3% | 1.0%  b A | 1.2%  b | 1.7%  ab A | 2.8%  a |
|  | Microascales | 2.9% | 1.2% | 0.5% | 0.5% | 1.2% | 1.6% | 0.7% | 1.4% | 0.7% | 0.9% | 0.6% | 1.0% |
| Basidiomycota | Tremellales | 2.5%  a | 3.4%  a | 1.3%  b AB | 1.0%  b | 2.5%  a | 2.7%  a | 1.1%  b B | 0.9%  b | 2.7% | 2.6% | 1.8%  A | 1.4% |
| Zygomycota | Mortierellales | 9.3%  ab | 11.7%  a | 5.9%  b B | 1.6%  c | 12.2%  a | 12.8%  a | 4.6%  b B | 1.5%  b | 12.5%  a | 10.1%  ab | 8.0%  b A | 3.2%  c |
|  | Mucorales | 8.1%  A | 9.5% | 5.4% | 10.8%  A | 1.4%  B | 2.2% | 2.0% | 3.6%  AB | 2.3%  B | 4.0% | 5.0% | 2.3%  B |

Different letters indicate significant differences (ANOVA, *P* < 0.05, Tukey’s HSD post-hoc analysis) among tillage (capital letter) and temporal-spatial treatments (small letter).
